# New insights into the mechanisms controlling the bronchial mucus balance

**DOI:** 10.1371/journal.pone.0199319

**Published:** 2018-06-22

**Authors:** Cyril Karamaoun, Benjamin Sobac, Benjamin Mauroy, Alain Van Muylem, Benoît Haut

**Affiliations:** 1 TIPs (Transfers, Interfaces and Processes), Université libre de Bruxelles, Brussels, Belgium; 2 Laboratoire J. A. Dieudonné, UMR CNRS 7351, Université Côte d’Azur, Nice, France; 3 Chest Department, Erasme University Hospital, Université libre de Bruxelles, Brussels, Belgium; Hospital for Sick Children, CANADA

## Abstract

In this work, we aim to analyze and compare the mechanisms controlling the volume of mucus in the bronchial region of the lungs of a healthy human adult, at rest and in usual atmospheric conditions. This analysis is based on a balance equation for the mucus in an airway, completed by a computational tool aiming at characterizing the evaporation, during respiration, of the water contained in the bronchial mucus. An idealized representation of the lungs, based on Weibel’s morphometric model, is used. The results indicate that the mechanisms controlling the volume of mucus in an airway depend on the localization of the airway in the bronchial region of the lungs. In the proximal generations, the volume of mucus in an airway is mainly controlled by the evaporation of the water it contains and the replenishment, with water, of the mucus layer by epithelial cells or the submucosal glands. Nevertheless, cilia beating in this part of the bronchial region remains of fundamental importance to transport the mucus and hence to eliminate dust and pathogens trapped in it. On the other hand, in the distal generations of the bronchial region, the volume of mucus in an airway is mainly controlled by the mucociliary transport and by the absorption of liquid by the epithelium. This absorption is a consequence of the mucus displacement by the cilia along generations with an interface between the epithelium and the airway surface layer of decreasing area. The numerical results obtained are in good agreement with previously published experimental data, thus validating our approach. We also briefly discuss how our results can improve the understanding and, possibly, the treatment of pulmonary diseases.

## Introduction

**Fig 1:**
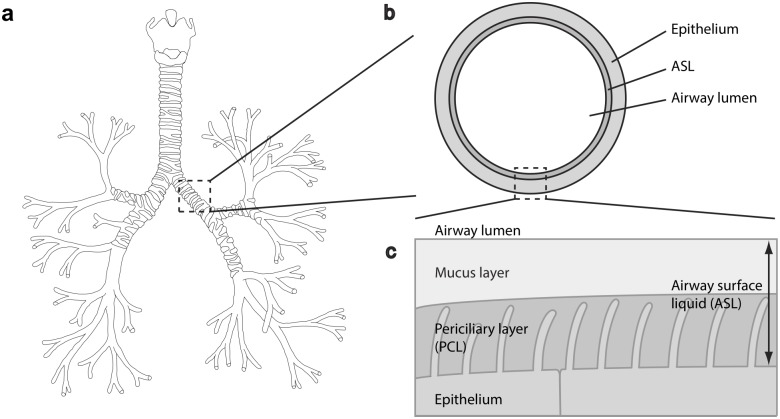
Three levels of details of the human bronchial region. **a**) Picture of a lung cast showing the human bronchial region. **b**) Schematic transverse view of an airway, with the different layers surrounding the lumen of the airway. **c**) The epithelial cilia penetrate in the ASL and generate the displacement of the mucus layer.

As the organ responsible for the respiration, the human lungs are dedicated to gas exchange between the body and the environment. The lungs form a dichotomous branching tree (see Fig 1a). Each level of subdivision of the tree structure is called a generation, the trachea being the first generation. In their simplest description, it is generally considered that the human lungs are composed of 24 generations and that they can roughly be divided in two regions: the bronchial region (generations 1 to 17), composed of airways (trachea, bronchi, bronchioles...) and designed for air transport, and the alveolar region (generations 18 to 24), composed of the alveoli and designed for respiratory gas exchange [1, 2].

The branching structure of the lungs allows creating a surface for gas exchange between the body and the environment with a large area. Actually, the lungs provide the largest surface of the human body in contact with the outside world (area between 50 and 100 m^2^) [3]. In order to protect this surface from particles and pathogens, most of the bronchial epithelium is covered with a protecting layer, called the Airway Surface Liquid (ASL) (see Fig 1b). This protecting layer is composed of two watery sublayers (see Fig 1c): the mucus layer, a thick gel-like substance made of water and large macromolecules (mucins), where foreign inhaled particles and pathogens are trapped in, and the Periciliary Layer (PCL), also a gel-like substance with a thickness of around 7 *μ*m, thought to serve as a lubricant for the mucus layer displacement [4, 5]. The epithelium is garnished by cilia (see Fig 1c) that contribute, by beating metachronously in the PCL, in displacing the mucus from the distal part of the bronchial region to the top of the trachea, where it is swallowed or expectorated. Therefore, this mucociliary transport (or clearance) constitutes a fundamental defense mechanism of the lungs against pathogens.

Major symptoms of several pulmonary diseases are correlated to an impairment of the mucociliary clearance, such as in chronic obstructive pulmonary disease (COPD) or, critically, in cystic fibrosis (CF) (see the review of Boucher [6]). For instance, Hogg et al. [7] have shown that accumulation of mucus in airway lumen is associated with progression of COPD. Regnis et al. [8] have shown that there is a mild defect in ciliary transport in human subjects with CF, when compared to controls. The defect correlates with their disease progression. Thus, a correct description of the bronchial mucus dynamics is crucial for the understanding of these diseases and for the development of new treatment strategies.

Several recent research works aimed at the analysis of the mucus dynamics, in fields such as molecular biology, biochemistry or rheology (see for instance the following reviews: [9, 10]). Among these studies, some dealt with mathematical modeling of the mucus dynamics, focusing on various aspects, ranging from the interaction of the mucus layer with the beating cilia [11, 12] to the regulation of the mucus chemical composition [13]. However, several aspects of the dynamics of the bronchial mucus still remain unclear. For instance, the different mechanisms controlling the volume of mucus in an airway are not clearly identified and characterized.

Four potential mechanisms controlling (actively or passively) the volume of mucus in an airway can be highlighted: the mucociliary transport, often mentioned as being the main mechanism of control [14], mass exchange between the mucus and the epithelium or the submucosal glands, the interaction between the mucus and the airflow in the lungs [15, 16] and the water exchange between mucus and inhaled or exhaled air (evaporation/condensation) [17].

In an airway, the ASL covers the apical surface of the epithelial cells (i.e. the surface of these cells facing the lumen). However, the total area of the apical surface of the epithelial cells in a generation increases with the generation index [1]. Hence, as the mucus is displaced by the cilia upwards the bronchial tree, it has less and less surface to spread on as it moves on. Therefore, as pointed out by several authors [18–20], this displacement could have a tendency to disrupt the balance of the mucus in the lungs. However, to the best of our knowledge, this point has never been thoroughly studied.

Regarding the mass exchange between the mucus and the epithelium, several works have shown that the epithelium is able to absorb liquid and that some cells of the epithelium have the ability to secrete mucus [14, 21]. It is also known that the submucosal glands are a significant source of mucus secretion in the first generations of the lungs [22–24].

It has long been believed that the upper respiratory tract is a perfect air conditioner (i.e. that, during an inhalation through the nose and at rest, the air at the entrance to the trachea is saturated with water vapor and at the body temperature) [18]. However, several experimental works have shown that this is not correct: the lower respiratory tract also acts substantially as an air conditioner [25–28]. For instance, McFadden et al. have shown that, in usual breathing conditions, the air can still be at a temperature lower than the body temperature even in the sixth generation [27]. In other words, these works show that air heating and water evaporation take place in the bronchial region of the lungs. As the air is only in contact with the mucus layer (if it is assumed that, in the generations where it is present, the ASL entirely covers the bronchial epithelium), this evaporation of water can only proceed from the mucus. Therefore, it causes a decrease of its volume and a reduction of its hydration level. In order to evaluate whether this effect is of significant importance on the control of the volume of mucus in an airway, a precise evaluation of the distribution, along the bronchial tree, of the evaporation rate of the water contained in the mucus is necessary. However, it should be highlighted that it has been shown that the temperature and the relative humidity of the inspired air have a significant influence on the efficiency of the mucociliary clearance [28–30]. As explained in more details later, these are indirect evidence of the fact that a significant evaporation of the water contained in the mucus can take place, at least in the first generations of the lungs.

In this work, we aim to give new insights into the mechanisms controlling the volume of mucus in the bronchial region of the lungs of a healthy human adult, at rest and in usual atmospheric conditions. First, a balance equation for the mucus in an airway is established and discussed. Then, the various potential mechanisms controlling the volume of mucus in an airway are analyzed and compared, using this balance equation, along the bronchial tree. In this context, the evaporation rate of the water contained in the mucus is evaluated in the different generations of the bronchial region, using a computational tool presented at the end of the paper. The results of this comparison provide a comprehensive scenario for the control of the mucus balance in the bronchial tree.

It must be emphasized that the approach followed in this work does not claim to provide highly predictive results. Indeed, as mentioned below, we are using an idealized geometrical representation of the lungs. Moreover, several physiological mechanisms involved are, to date, still partially characterized. However, special attention is paid in this article to the collection of reliable data and to a discussion of the assumptions made. This provides results that make it possible to comprehend what are the different phenomena controlling the volume of mucus in an airway and to give orders of magnitude of their amplitude.

## Geometrical representation of the lungs and parameters of the study

**Fig 2:**
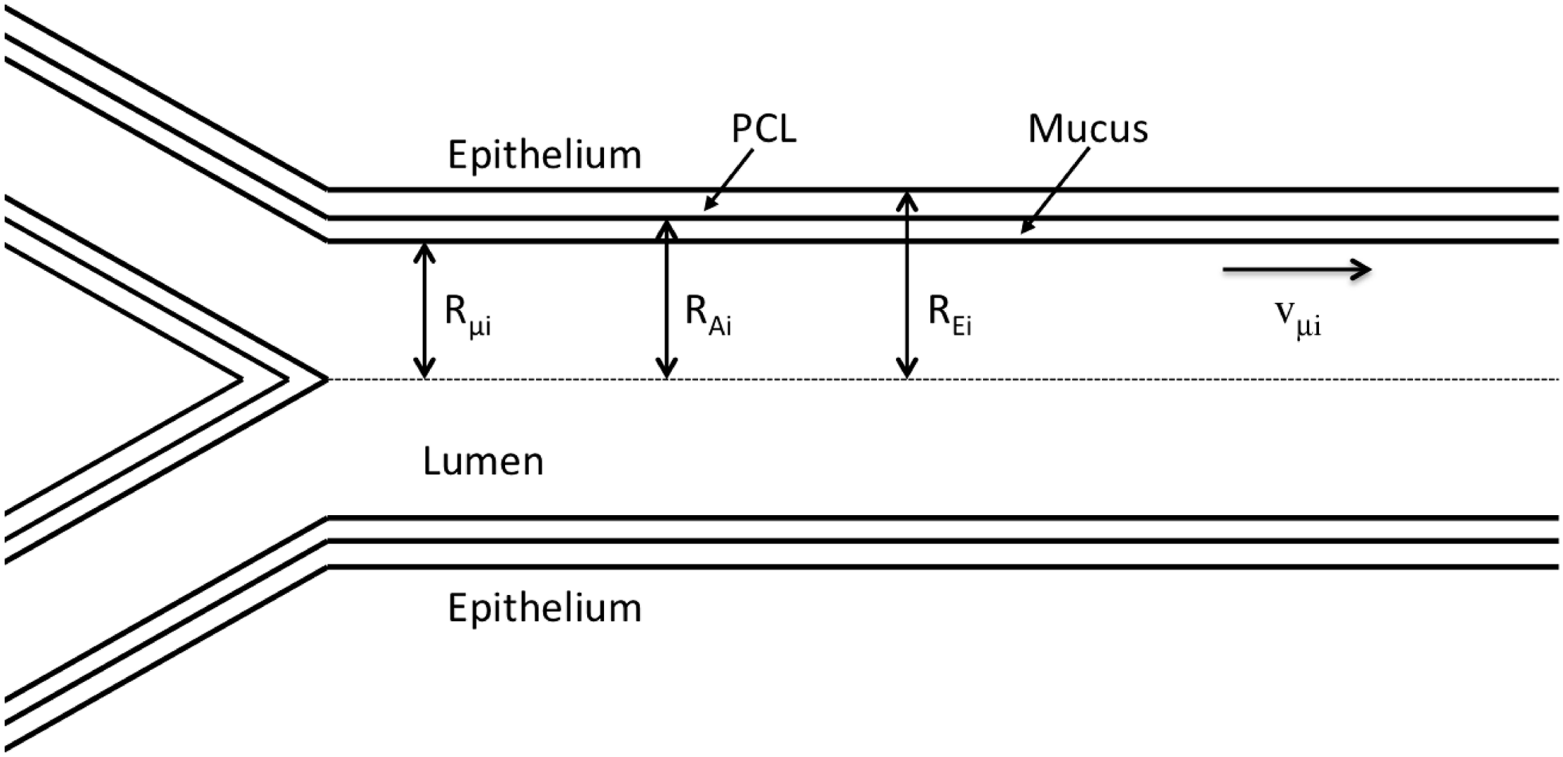
Scheme of an airway in generation *i* and a bifurcation. Several parameters related to the balance of the mucus in the airway are represented on this figure. The picture is not to scale.

In this work, we use a idealized geometrical representation of the human lungs extensively described in a previous paper [31]. It is based on Weibel’s morphometric model [1]. This model considers that all the airways are right circular cylinders and that all the airways belonging to the same generation have the same dimensions. Each airway belonging to generation *i* is characterized by one length, *L*_*i*_ (m), and one airway diameter, *R*_*E*_*i*__ (m). It divides in two airways belonging to generation *i* + 1. This division is called a bifurcation. It is worth pointing out that we conducted the same analysis with another simplified geometry of the lungs, also conventionally encountered in the literature, and defined by the following relations: *L*_*i*+1_ = 2^−1/3^ *L*_*i*_, *R*_*E*_*i*+1__ = 2^−1/3^ *R*_*E*_*i*__, *R*_*E*_*i*__/*L*_*i*_ = 1/3 and *R*_*E*_1__ = 1 cm. Interestingly, the results obtained are very close to those presented in this article and they are qualitatively the same. As sketched in Fig 2, it is assumed that, in the generations where it is present, the ASL entirely covers the bronchial epithelium.

Several parameters used in this study and their values are presented in Table 1. The values of *δ*_*μ*_, *f*, *Q*^in^, *t*^in^ and *v*_*μ*_ given in this table are typical of a healthy adult, at rest. The range of values of RH^in^ and *T*^in^ given in the table are typical ranges of values of the relative humidity and the temperature of the air at the inlet of the trachea, during inspiration, at rest and in usual atmospheric conditions (from outdoor cold and dry air to normal indoor air) [25, 27, 32].

**Table 1:** Values of several parameters used in this work.

Parameter	Notation	Value	Units	References
Mucus layer thickness in the trachea	*δ*_*μ*_	10-30	*μ*m	[12, 33–35]
Mucus layer thickness in distal generations	*δ*_*μ*_	2-5	*μ*m	[34, 36]
Respiration frequency	*f*	0.25	s^−1^	[3]
Relative humidity of the air at the inlet of the trachea, during inspiration	RH^in^	0.80-0.95	-	[25, 27, 32]
Inspiration flow rate	*Q*^in^	250	ml s^−1^	[3]
Inspiration time	*t*^in^	2	s	[3]
Temperature at the inlet of the trachea during inspiration	*T*^in^	30-34	°C	[25, 27, 32]
Temperature of the body	*T*_b_	37	°C	
Mucus velocity due to cilia beating in the trachea	*v*_*μ*_	5	mmmin^−1^	[37–40]

## Results and discussion

### Mucus balance in an airway

At a time scale way larger than the duration of a respiratory cycle, at rest, in usual atmospheric conditions and in the absence of a pathology, it can be assumed that the total volume of mucus in an airway is constant. If it was not the case, a total depletion of the mucus or an obstruction of the airway would eventually occur. Accordingly, a balance equation for the mucus in an airway of generation *i* can be written as follows (see Fig 2):

$$\begin{array}{c} 2\pi \left(R_{A_{i+1}}^{2}-R_{\mu_{i+1}}^{2}\right)v_{\mu_{i+1}}-\pi \left(R_{A_{i}}^{2}-R_{\mu_{i}}^{2}\right)v_{\mu_{i}}-E_{i}+B_{i}=0 \end{array}$$ (1)

where *v*_*μ*_*i*__ is the average velocity of the mucus due to cilia beating in generation *i* (ms^−1^), *R*_*A*_*i*__ is the distance between the center of an airway of generation *i* and the interface between the PCL and the mucus layer (m), *R*_*μ*_*i*__ is the distance between the center of an airway of generation *i* and the interface between the mucus layer and the lumen (m), *E*_*i*_ is the volume of water evaporated in an airway of generation *i* during a respiratory cycle, divided by the duration of the cycle (m^3^ of liquid water per second), and *B*_*i*_ is a term accounting for the other potential mechanisms controlling the mucus volume in an airway of generation *i* (m^3^s^−1^). For instance, the mass exchange mechanisms between the mucus layer and the epithelium or the submucosal glands are included in *B*_*i*_. This term is positive if these mechanisms tend to increase the volume of mucus in the airway.

The first term of the left-hand side of this equation is the volume of mucus brought, per unit time, in the airway by the cilia beating from its two daughters. The second term of the left-hand side of this equation is the volume of mucus leaving the airway per unit time, again by cilia beating.

In healthy people, the thickness of the PCL is close to 7 *μ*m, the thickness of the mucus layer is between 2-5 *μ*m in distal generations and 10-30 *μ*m in the trachea (see next section), while the lumen of the airways has a radius of around 250 *μ*m in distal generations and 1 cm in the trachea [1]. Therefore, in healthy people, it can be assumed that, in any generation of the bronchial region of the lungs, the mucus layer thickness in an airway, *δ*_*μ*_*i*__ = *R*_*A*_*i*__ − *R*_*μ*_*i*__ (m), and the thickness of the PCL are way smaller than the radius of the airway lumen. Therefore, *R*_*μ*_*i*__ ≃ *R*_*A*_*i*__ ≃ *R*_*E*_*i*__ (m), the distance between the center of the lumen and the epithelium–PCL interface, in generation *i* (see Fig 2). Accordingly, Eq (1) can be rewritten:

$$\begin{array}{c} 4\pi R_{E_{i+1}}\delta_{\mu_{i+1}}v_{\mu_{i+1}}-2\pi R_{E_{i}}\delta_{\mu_{i}}v_{\mu_{i}}-E_{i}+B_{i}=0 \end{array}$$ (2)

As described by Weibel et al., the relation between the radii of airways in two successive generations can be approximated by *R*_*E*_*i*+1__ = $2^{-\frac{1}{3}}\;R_{E_{i}}$ [2]. Using this relation, Eq (2) can be rewritten:

$$\begin{array}{c} 2\pi R_{E_{i}}\delta_{\mu_{i}}v_{\mu_{i}}\left(2^{\frac{2}{3}}\frac{\delta_{\mu_{i+1}}v_{\mu_{i+1}}}{\delta_{\mu_{i}}v_{\mu_{i}}}-1\right)-E_{i}+B_{i}=0 \end{array}$$ (3)

If *E*_*i*_ and *B*_*i*_ were both equal to zero, the balance equation for the mucus in an airway of generation *i* would be written:

$$\begin{array}{c} \delta_{\mu_{i}}v_{\mu_{i}}=2^{\frac{2}{3}}\delta_{\mu_{i+1}}v_{\mu_{i+1}}\approx 1.6\;\delta_{\mu_{i+1}}v_{\mu_{i+1}} \end{array}$$ (4)

Eq (4) shows that, if *E*_*i*_ and *B*_*i*_ were both equal to zero, *δ*_*μ*_ *v*_*μ*_, the product of the mucus layer thickness and the average velocity of the mucus (due to cilia beating), would have to be multiplied by a factor of 1.6 over the generations *i* + 1 and *i* to ensure the mucus balance in the generation *i*. This increase of *δ*_*μ*_ *v*_*μ*_ is needed to compensate the reduction of the total area of the epithelium–ASL interface in the airways of generation *i*, when compared to generation *i* + 1. Considering that the maximal extent of the mucus layer is up to generation 16 [15], the magnitude of the increase of *δ*_*μ*_ *v*_*μ*_ over the whole bronchial tree should be about $2^{\frac{2\times 15}{3}}\approx 10^{3}$ to ensure the mucus balance in the entire bronchial region, if *E*_*i*_ and *B*_*i*_ were both equal to zero everywhere in this region. As discussed later, this seems unlikely, according to results presented in previous works. Therefore, the balance of the mucus appears difficult without the existence of mechanisms controlling the volume of mucus in an airway other than the cilia beating, i.e. *B*_*i*_ and *E*_*i*_ should not be both equal to zero over the whole bronchial region of the lungs.

Note that, rigorously, for Eq (1) to be correct, *R*_*μ*_*i*__ should have been defined as the distance between the center of an airway of generation *i* and the interface between the mucus layer and the lumen, at the opening of the airway (i.e. at the entrance of the air in the airway during inspiration). A similar comment should be made about *v*_*μ*_*i*__. However, this abuse of terminology does not have any influence on the results presented in this paper.

### Scale analysis of the mechanisms controlling the balance of the bronchial mucus

In this section, the various mechanisms potentially controlling the volume of mucus in an airway are further analyzed.

#### Mucociliary transport

Mucociliary transport is usually reported as being the main mechanism controlling the bronchial mucus balance. Numerous references indicate that, in the trachea, the average velocity of the mucus is close to 5 mm min^−1^ and the thickness of the mucus layer is about 10 *μ*m (even if higher values, up to 30 *μ*m, are also reported) [12, 33–35, 37–40]. For distal generations, it is usually considered that the thickness of the mucus layer is about 2-5 *μ*m [34, 36]. However, there is little information in the literature regarding the average velocity of the mucus in these generations. By modeling, some authors have calculated that the average velocity of the mucus in distal generations should be three orders of magnitude smaller than the velocity in the trachea [41]. However, these results were obtained by neglecting, in the mucus balance equation in an airway, all the mechanisms other than cilia beating (i.e. the authors performed the reasoning given in the previous section, but setting immediately *E*_*i*_ and *B*_*i*_ both equal to zero), whereas results presented later show that, for example, *E*_*i*_ can usually not be neglected in the balance equation for an airway in generations 1 to 8. Moreover, experimental data reported for the dog and the rat [18, 42] show that the average velocity of the mucus in the distal generations is approximately one order of magnitude smaller than the velocity in the trachea. Therefore, it seems more realistic to rely on these data to characterize the velocity of the mucus in the bronchial region of the human lungs. As a consequence, we can calculate that the transport rate of the mucus in the bronchial region of the human lungs (*δ*_*μ*_ *v*_*μ*_) is expected, when going from the distal to the proximal generations, to increase by approximately a factor of 50 (considering *δ*_*μ*_1__ = 10 *μ*m, *v*_*μ*_1__ = 5 mm min^−1^, *δ*_*μ*_16__ = 2 *μ*m and *v*_*μ*_16__ = 0.5 mm min^−1^). When compared to the analysis presented in the previous section, this calculation shows clearly that the mucociliary transport cannot be the only mechanism controlling the balance of the mucus in the bronchial region of the lungs.

According to the simplifications mentioned previously, the volume of mucus leaving the trachea per unit time due to cilia beating can be evaluated as follows: ${\dot{V}}_{\mu }=2\pi R_{E_{1}}\delta_{\mu_{1}}v_{\mu_{1}}$. Using *R*_*E*_1__ = 7.6 mm [31], *δ*_*μ*_1__ = 10 *μ*m and *v*_*μ*_1__ = 5 mm min^−1^, ${\dot{V}}_{\mu }=0.04$ *μ*l s^−1^ is calculated.

#### Evaporation

In order to quantitatively analyze the evaporation of the water contained in the mucus in the bronchial region of the lungs of a healthy human adult (term *E*_*i*_ in Eq (1)), we have developed a computational tool, fully described in the last part of this paper. This computational tool is based on the expression of mass and energy transport equations in the lumen of the airways as well as in the ASL and the epithelium. An original feature of this computational tool relies in an energy balance equation for the connective tissue surrounding the airway walls. This equation allows calculating the temperature of this tissue, by expressing that it is controlled by the flow of energy towards the lumen (to ensure the heating of the air and the evaporation of the water) and by the blood flow in the tissue.

The analysis of the evaporation of the water contained in the mucus and, more generally, of the heat and water vapor transport in the bronchial region of the lungs have been the subject of several previous works [21, 43–47]. However, these studies mainly focused on the calculation of the air temperature in this bronchial region or on the calculation of the water vapor content of the exhaled air. The published results do not provide complete information about the distribution of the evaporation flux density in the lungs or about the temperature of the mucus. Consequently, we have developed our own computational tool, which is able to predict the distribution in the lungs of the physical quantities that are pertinent for our study. Of course, we have compared the results obtained with our computational tool to these other computational approaches and they are very similar.

**Fig 3:**
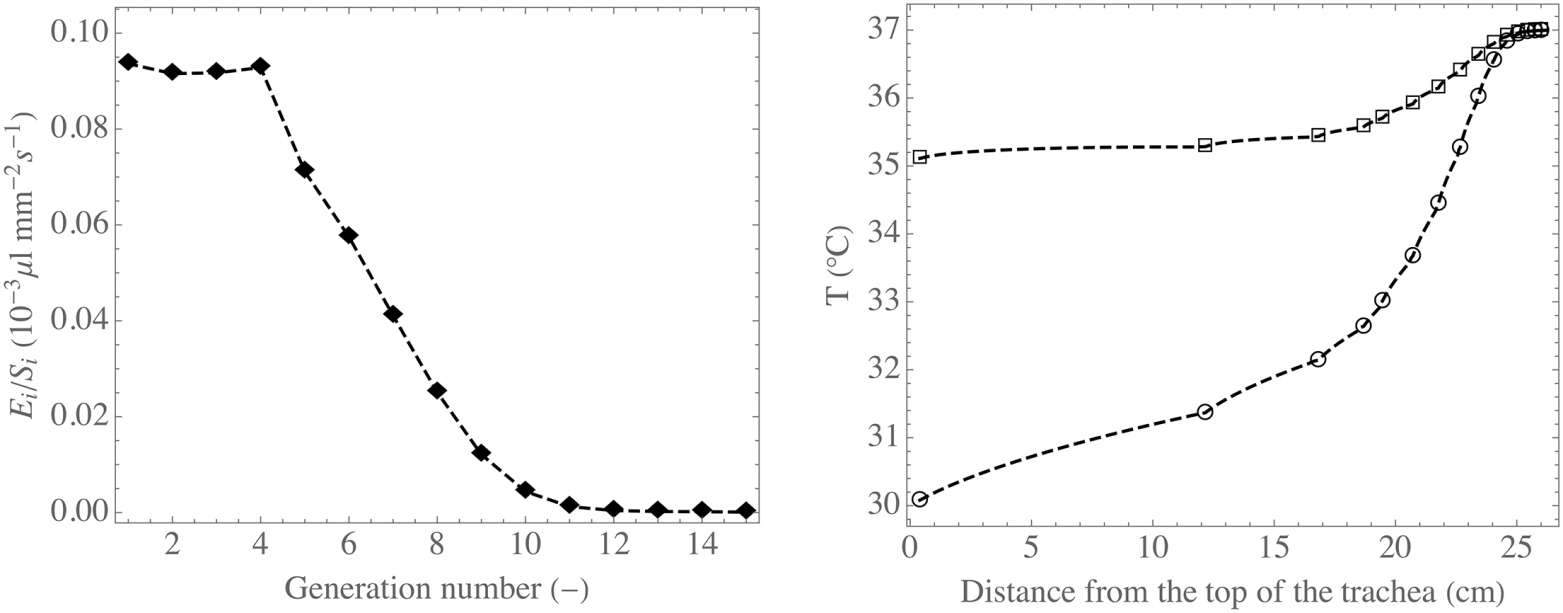
Results obtained with the computational tool. Left: calculated value of *E*_*i*_/*S*_*i*_ as a function of the generation index. Right: calculated average temperature of the air on a flow cross-section as a function of the distance from the top of the trachea, at the end of the inspiration (circles) and the expiration (squares). These results have been obtained with the computational tool, using *t*^in^ = 2 s, *f* = 0.25 s^−1^, *Q*^in^ = 250 ml s^−1^, *T*^in^ = 30°C and RH^in^ = 0.80.

For given respiratory conditions (*Q*^in^, *t*^in^, *T*^in^, RH^in^, *f*), the computational tool first calculates the temperature and the water vapor concentration fields in the bronchial region of the lungs, at any time during a respiratory cycle. Then, from these calculated fields, it evaluates *E*_*i*_ for each value of *i*. Then, if *S*_*i*_ is defined as the area of the interface between the epithelium and the ASL in an airway of generation *i*: *S*_*i*_ = 2*πR*_*E*_*i*__ *L*_*i*_, the evaporation flux density in each generation, *E*_*i*_/*S*_*i*_, is calculated. In Fig 3 (left), calculated values of *E*_*i*_/*S*_*i*_ are presented. They have been computed with *t*^in^ = 2 s, *f* = 0.25 s^−1^, *Q*^in^ = 250 ml s^−1^, *T*^in^ = 30°C and RH^in^ = 0.80.

Fig 3 (left) reveals that the value of *E*_1_/*S*_1_ is close to 0.10 × 10^−3^ *μ*l s^−1^ mm^−2^. Therefore, taking *L*_1_ = 12 cm and *R*_*E*_1__ = 7.6 mm [31], *E*_1_ ≃ 0.6 *μ*l s^−1^ is calculated, for the conditions used to generate Fig 3. The comparison of this value of *E*_1_ and the value of ${\dot{V}}_{\mu }$ calculated previously clearly shows that the evaporation is far from being negligible in controlling the mucus balance in the bronchial region of the lungs, at least in the first generations.

Widely referenced experimental results regarding heat transfer in the lungs are those of McFadden et al. [27]. For different inspiration and expiration flow rates and for different types of ambient air, these authors measured the air temperature in the lumen of the bronchi, at the end of the inspiration and at the end of the expiration, down to the sixth generation. In particular, they showed that, during expiration, the air is significantly cooled when it flows from the alveolar region of the lungs to the trachea. In Fig 3 (right), results obtained with the computational tool are presented. This figure shows the calculated average temperature of the air on a flow cross-section, as a function of the distance from the top of the trachea, at the end of the inspiration (circles) and the expiration (squares). As it can be observed, the computational tool is able to simulate the cooling of the air during expiration. The analysis of the simulation results allows highlighting that it is a consequence of the fact that, during inspiration and in the first generations of the lungs, the blood flow in the connective tissue is not able to significantly offset the loss of energy due to air heating and evaporation of water; it results in a significant decrease of the temperature of this tissue. Subsequently, energy is transfered back from the air to the connective tissue during expiration, leading to condensation of water, since it is calculated that the air is at the body temperature and saturated with water when it leaves the alveolar region. The analysis of the simulation results also highlights that this condensation has a significant influence on *E*_*i*_.

#### Other mechanisms controlling the mucus volume in an airway

Two other mechanisms possibly controlling the volume of mucus in a generation can be listed: the interaction between the airflow and the mucus layer and the mass exchange between the mucus layer and the epithelium or the submucosal glands.

Regarding the interaction between the airflow and the mucus layer, recent studies have shown that the airflow in the airways is able, in some conditions, to displace the mucus [15, 16]. Actually, the airflow applies a stress on the mucus layer; if this stress is high enough, the airflow is able to drag the mucus and to make it move. This is typically what happens during cough or chest physiotherapy. This interaction has been studied by Mauroy et al. [15]. Their mathematical model notably predicts, in generation *i*, the threshold thickness *δ*_*μ*_max,*i*__ of the mucus layer under which it is unaffected by an airflow of a given mean superficial velocity Φ_*i*_ (m s^−1^). Φ_*i*_ is defined as the flow rate in the generation divided by the total area of the flow cross-section, in the absence of the ASL. *δ*_*μ*_max,*i*__ depends also on the airway radius *R*_*E*_*i*__, on the air viscosity *μ*_*a*_ (kg m^−1^ s^−1^) and on the stress threshold *σ*_0_ (kg m^−1^ s^−2^) needed to be overcome in the mucus for making it flow (the mucus is considered as a threshold Bingham fluid):

$$\begin{array}{c} \frac{\delta_{\mu_{\text{max},i}}}{R_{E_{i}}}=1-{\left(\frac{4\mu_{a}\Phi_{i}}{R_{E_{i}}\sigma_{0}}\right)}^{\frac{1}{4}} \end{array}$$ (5)

Using *R*_*E*_*i*+1__ = $2^{-\frac{1}{3}}\;R_{E_{i}}$ and $\Phi_{i}=Q^{\text{in}}/\left(2^{i-1}\pi R_{E_{i}}^{2}\right)$, the previous equation can be rewritten as:

$$\begin{array}{c} \delta_{\mu_{\text{max},i}}=\frac{1}{2^{\frac{i-1}{3}}}\left(R_{E_{1}}-{\left(\frac{4\mu_{a}Q^{\text{in}}R_{E_{1}}}{\pi \sigma_{0}}\right)}^{\frac{1}{4}}\right) \end{array}$$ (6)

This equation shows that *δ*_*μ*_max,*i*__ is a monotonically decreasing function of *i*. The application of this equation with *μ*_*a*_ = 1.9 × 10^−5^ kg m^−1^ s^−1^, Q^in^ = 250 ml s^−1^, *R*_*E*_1__ = 7.6 mm and *σ*_0_ = 0.1 kg m^−1^ s^−2^ [15] gives *δ*_*μ*_max,1__ = 3 mm and *δ*_*μ*_max,16__ = 90 *μ*m. When compared to the data provided in Table 1, these results show that, for a healthy human adult at rest, the airflow in the bronchial region of the lungs is not likely to interact with the mucus layer, even in the smallest airways. In other words, the interaction between the airflow and the mucus layer does not appear to be a mechanism possibly controlling the volume of mucus in a generation, for healthy people at rest.

Regarding the mass exchange between the mucus layer and the epithelium or the submucosal glands, several authors observed the existence of a physiological mechanism of control of the water content of the mucus layer [13, 20, 21, 46, 48]. Although not fully understood, this mechanism seems to imply a regulation of the ionic balance in the ASL, controlling its thickness and its water balance [48]. Several studies have reported absorption rates of the mucus by the epithelium between 0.013 × 10^−3^ and 0.025 × 10^−3^ *μ*l s^−1^ mm^−2^ [21]. *In vitro* experiments have shown that bronchial epithelium cells can replenish a mucus layer covering them with up to 0.3 × 10^−3^ *μ*l of water per second and per square millimeter of the epithelium–ASL interface [20, 46]. Interestingly, the values of *E*_*i*_/*S*_*i*_ presented in Fig 3 have the same order of magnitude, in the first generations. It indicates that water replenishment of the mucus layer by the epithelium is a mechanism that can significantly control the mucus balance, at least in the first generations of the lungs.

### Distribution, along the bronchial tree, of the amplitude of the mechanisms controlling the volume of mucus in an airway

The scale analysis presented in the previous section shows that the mucociliary transport, the evaporation of the water contained in the mucus and the mass exchange between the mucus layer and the epithelium or the submucosal glands (corresponding to the term *B*_*i*_ in Eq (1)) can have a significant influence on the volume of mucus in an airway.

**Fig 4:**
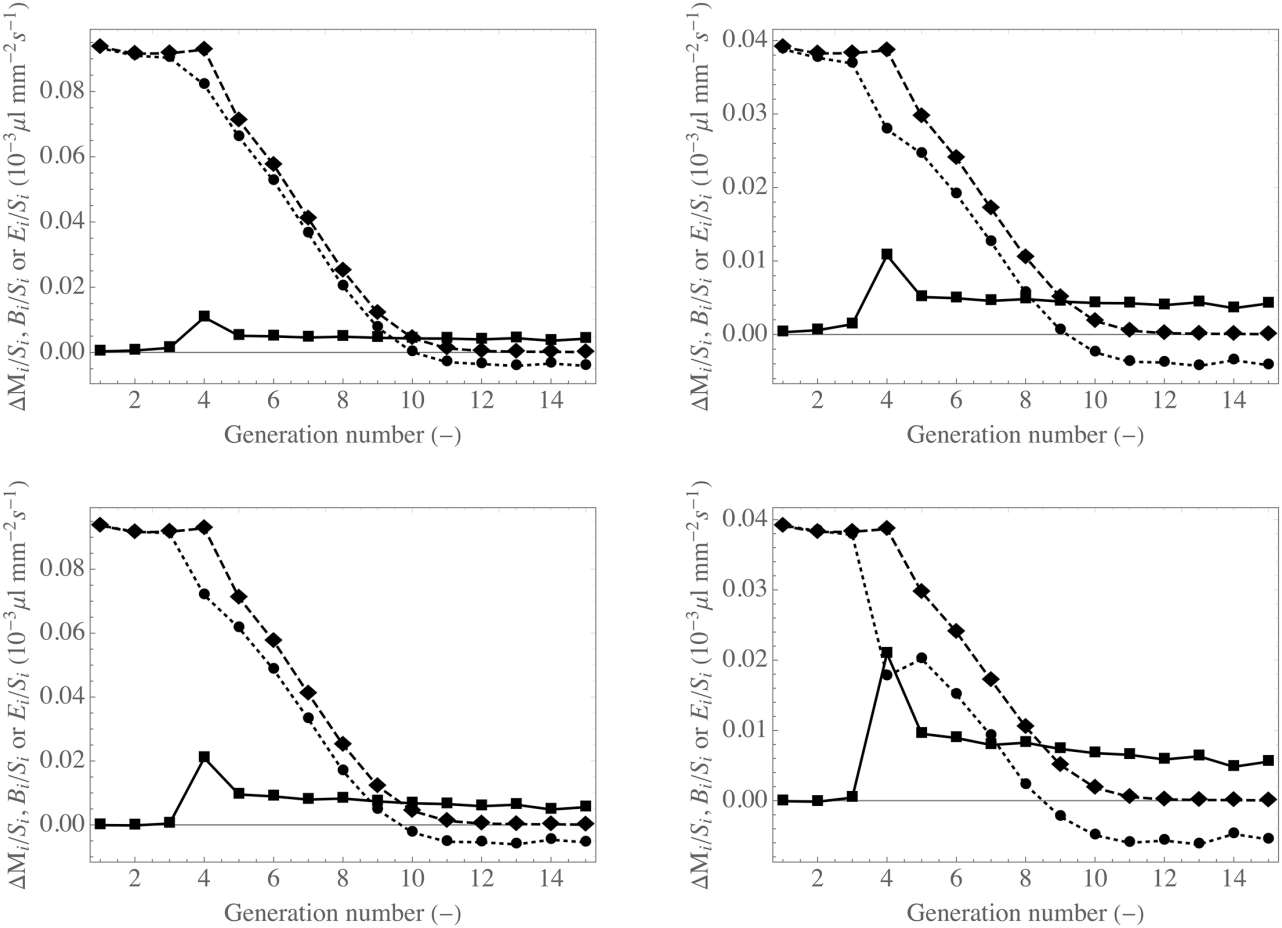
Magnitude of the various mechanisms controlling the bronchial mucus balance. The results were obtained with the data given in Table 1. Left figures: *T*^in^ = 30°C and RH^in^ = 0.80, right figures: *T*^in^ = 34°C and RH^in^ = 0.95. Top figures: *δ*_*μ*_1__ = 10 *μ*m, *v*_*μ*_1__ = 5 mm min^−1^, *δ*_*μ*_16__ = 2 *μ*m and *v*_*μ*_16__ = 0.5 mm min^−1^. Bottom figures: *δ*_*μ*_1__ = 30 *μ*m, *v*_*μ*_1__ = 5 mm min^−1^, *δ*_*μ*_16__ = 3 *μ*m and *v*_*μ*_16__ = 0.5 mm min^−1^. Squares-continuous lines: Δ*M*_*i*_, diamonds-dashed lines: *E*_*i*_, circles-dotted lines: *B*_*i*_.

In Fig 4, *E*_*i*_/*S*_*i*_, Δ*M*_*i*_/*S*_*i*_ and *B*_*i*_/*S*_*i*_ are presented, for *i* = 1, .., 15. As mentioned previously, *S*_*i*_ = 2*πR*_*E*_*i*__ *L*_*i*_ is the area of the epithelium–ASL interface in an airway of generation *i*. Δ*M*_*i*_ is defined as the difference between the volume of mucus entering, per unit time, an airway of generation *i* due to cilia beating and the volume of mucus leaving, per unit time, this airway due to cilia beating. According to previous notations and assumptions, Δ*M*_*i*_ can be expressed as: Δ*M*_*i*_ = 4*πR*_*E*_*i*+1__ *δ*_*μ*_*i*+1__ *v*_*μ*_*i*+1__ − 2*πR*_*E*_*i*__ *δ*_*μ*_*i*__ *v*_*μ*_*i*__. Following Eq (1), *B*_*i*_ = *E*_*i*_ − Δ*M*_*i*_. The data presented in Table 1 have been used to compute the data presented in this figure (left figures: *T*^in^ = 30°C and RH^in^ = 0.80, right figures: *T*^in^ = 34°C and RH^in^ = 0.95).

According to the results mentioned previously, two probable scenarios are considered to calculate the values of Δ*M*_*i*_ presented in Fig 4. On the top figures, a geometric progression for *δ*_*μ*_*i*__ *v*_*μ*_*i*__ is used (i.e. *δ*_*μ*_*i*+1__ *v*_*μ*_*i*+1__ = (1/*k*)*δ*_*μ*_*i*__ *v*_*μ*_*i*__), with *δ*_*μ*_1__ = 10 *μ*m, *v*_*μ*_1__ = 5 mm min^−1^, *δ*_*μ*_16__ = 2 *μ*m and *v*_*μ*_16__ = 0.5 mm min^−1^. On the bottom figures, another geometric progression is used, with *δ*_*μ*_1__ = 30 *μ*m, *v*_*μ*_1__ = 5 mm min^−1^, *δ*_*μ*_16__ = 3 *μ*m and *v*_*μ*_16__ = 0.5 mm min^−1^. The hypothesis of a geometric progression to describe the evolution of lung properties along the bronchial tree is common [49]. For instance, Yager et al. have shown that it is valid for describing the mucus thickness in Guinea pigs [36]. Nevertheless, we have also tested a linear decay scenario for *δ*_*μ*_ *v*_*μ*_ and the results obtained are qualitatively the same.

Several interesting conclusions can be drawn from the analysis of the results presented in Fig 4.

The evaporation of the mucus mainly occurs in the proximal part of the bronchial region of the lungs. *E*_*i*_/*S*_*i*_ is almost constant in the four first generations and then monotonically decreases when the generation index increases. *E*_*i*_/*S*_*i*_ is almost equal to zero starting from generation 10. Therefore, when the inspired air reaches generation 10, it is almost saturated with water and at body temperature.

The results presented in Fig 4 indicate that the mechanisms controlling the volume of mucus in an airway are different depending on whether the airway is in a proximal or a distal generation.

In the proximal generations of the bronchial region, the results presented in Fig 4 suggest that the volume of mucus in an airway is mainly controlled by the evaporation of water and the replenishment, with water, of the mucus layer by the epithelium and the submucosal glands. Indeed, in these generations, *E*_*i*_ is way larger than Δ*M*_*i*_. Accordingly, *B*_*i*_ is positive; it corresponds to a replenishment of the mucus with water. Interestingly, it is known that the submucosal glands are precisely abundant in the first generations of the lungs only [22–24]. Of course, cilia beating in this part of the bronchial region remains of fundamental importance to transport the mucus and hence to eliminate dust and pathogens trapped in the mucus layer.

On the other hand, in the distal generations of the bronchial region, the results presented in Fig 4 suggest that the volume of mucus in an airway is mainly controlled by the mucociliary transport and by the absorption of liquid by the epithelium (*B*_*i*_ < 0). Interestingly, the calculated values of *B*_*i*_/*S*_*i*_ in these generations have the same order of magnitude than absorption rates of liquid by the epithelium determined experimentally and reported by several studies (see previous section) [21]. However, it should be borne in mind that the calculated values of *B*_*i*_ are intimately related to the distribution chosen for the product *δ*_*μ*_*i*__ *v*_*μ*_*i*__ within the bronchial tree. The results obtained are to be taken with caution, as they are based on several modeling assumptions that need to be either confirmed or refined. They indicate that an absorption of liquid by the epithelium is very probable in distal generations. It is interesting to note that Asmundsson et al. have observed that, in dog lungs, the mucus is more watery in distal generations than in proximal ones [18]. The authors conclude that this probably highlights an absorption of water by the epithelium when the mucus is moved along distal generations.

In intermediate generations, the results presented in Fig 4 show that the mucus volume in an airway is controlled by a mix of different mechanisms, depending on the breathing conditions and on the scenario chosen for the evaluation of *δ*_*μ*_*i*__ *v*_*μ*_*i*__.

### Perspectives regarding the understanding of pulmonary diseases

In CF patients, there is an impairment of the mucus hydration [9, 22–24]. Our results give a straightforward explanation on why this could lead to a mucus with increased viscosity. Indeed, as a consequence of the impaired hydration, the value of the replenishment term, *B*_*i*_, in the first generations of the lungs is reduced in case of CF. On the other hand, except in the case of a severe obstruction of the airways and/or in the case of a mucus with a greatly increased amount of mucins, the values of the evaporation rate, *E*_*i*_, should not be significantly impacted in case of CF. Moreover, the mucins are of course not evaporated during respiration. As evaporation and mucus replenishment appear to be the two mechanisms controlling the volume of mucus in the first generations of the lungs, this water unbalance will eventually lead to an increased molar fraction of mucins in the first generations and, hence, to an increased viscosity of the mucus in these generations. This in turn would make the mucus difficult to move by the cilia. However, it is important to note this may be partly offset by the fact that an increase of the mucus thickness due to its reduced mobility can induce an interaction between the airflow and the mucus, leading to a significant displacement of it. This effect is, however, difficult to quantify precisely.

One of the treatment proposed to ease the symptoms of CF is chest physiotherapy. It helps the CF patients to evacuate the excess of mucus from their airways [50]. It is known that physiotherapy maneuvers are facilitated by having the patients inhaling a hypertonic saline solution. It has been shown that this solution improves the mucociliary clearance by creating an osmotic transfer of water from the epithelium to the ASL (increase of the replenishment term *B*_*i*_) [51], contributing to the recovery of the volume and the rheological properties of the mucus [52]. Our results support the assumption that this effect could be mimicked or amplified by having the patients breathing in a conditioned air (with high temperature and RH). Indeed, for instance, the evaporation in the first generations can be totally canceled or even reversed (to condensation) by breathing an air saturated with water and with a temperature larger than the body temperature. As evaporation is a mechanism significantly controlling the water content of the mucus layer in the first generations (and tending to decrease this water content), this could contribute to have a mucus with a significantly increased water content and, hence, easier to evacuate due to its decreased viscosity. This should of course be confirmed experimentally, although the measurement of the efficiency of chest physiotherapy is still a challenging task [53].

The calculation results presented in Fig 4 show that, at rest, the breathing of cold and dry air significantly increases (by more than a factor of two) the evaporation rate of the water contained in the mucus of proximal generations, when compared to the breathing of indoor air. Using the computational tool, it is possible to show that, if exercise is performed in cold and dry air, this evaporation rate could even be increased by a factor of ten, when compared to an indoor situation, at rest. Since evaporation is a mechanism of considerable importance for the control of the mucus volume in a proximal airway, it is understood that breathing cold and dry air can have a significant effect on the degree of mucus hydration in such an airway (and therefore on the viscosity/mobility of the mucus in the airway). In addition, since evaporation is very heat-intensive (it is calculated that it requires an amount of heat about 10 times more than the heat required for air heating), the inspiration of cold and dry air can significantly lower the temperature of the connective tissue around the airways in the proximal generations. When performing exercises in cold and dry air, the calculations show that this temperature can even be lowered to values of 30°C, in a healthy person, because of the inability of the vascularization of the tissue to bring in enough heat to compensate for this evaporation. These results provide interesting insights: some forms of asthma involve a lack of vascularization of the connective tissue surrounding airways [54] and people with asthma are very prone to bronchoconstriction when they breathe cold and dry air [55]. In addition, these results are also consistent with the fact that breathing continuously cold air increases the risk of infection of the lower respiratory tract [56].

## Conclusion

In this work, a new analysis of the mechanisms controlling the mucus balance in the bronchial region of the lungs is presented.

A scale analysis shows that, in an airway, the mucus transport by cilia beating, the evaporation of water contained in the mucus and the mass exchange between the mucus layer and the epithelium or the submucosal glands are three mechanisms that can be of significant importance in the control of the volume of mucus in the airway. More precisely, the coupling of the balance equation and our computational tool indicate that the mechanisms controlling the volume of mucus in an airway depend on the localization of the airway in the bronchial region of the lungs.

In the proximal generations, our results suggest that the volume of mucus in an airway is mainly controlled by the evaporation of water and the replenishment, with water, of the mucus layer by the epithelium. On the other hand, in the distal generations of the bronchial region, our results suggest that the volume of mucus in an airway is mainly controlled by the mucociliary transport and possibly by the absorption of liquid by the epithelium. It is also worth mentioning that the good comparison between the calculated values of *B*_*i*_/*S*_*i*_ and the experimental results presented in previous works supports the validity of the approach followed in this paper.

The present work aims at giving new information regarding the control of the bronchial mucus balance, which is critical in several pulmonary diseases, such as cystic fibrosis or chronic obstructive pulmonary diseases. Altogether, our results indicate that the understanding of the dynamics of the bronchial mucus could help to develop new or adapted treatment strategies for such diseases. The present analysis should be compared to additional experimental data, especially regarding the magnitude of the identified control mechanisms such as the replenishment of the mucus layer or the absorption of liquid by the epithelium. The mathematical approach developed in this work could also be further refined, if additional physiological data related to the bronchial mucus dynamics would become available.

In this work, we considered the case of a human adult, at rest and under normal breathing conditions. This is a reference situation. However, in the future, we intend to use our approach to investigate the bronchial mucus balance in other conditions (e.g. respiration in infants, exercise in cold/dry air conditions, presence of ciliary abnormalities, tracheostomy placement...). But it should be kept in mind that going beyond this reference situation might require physiological information that are not necessary easy to obtain.

## Computational tool for the characterization of the evaporation of the water contained in the mucus

**Fig 5:**
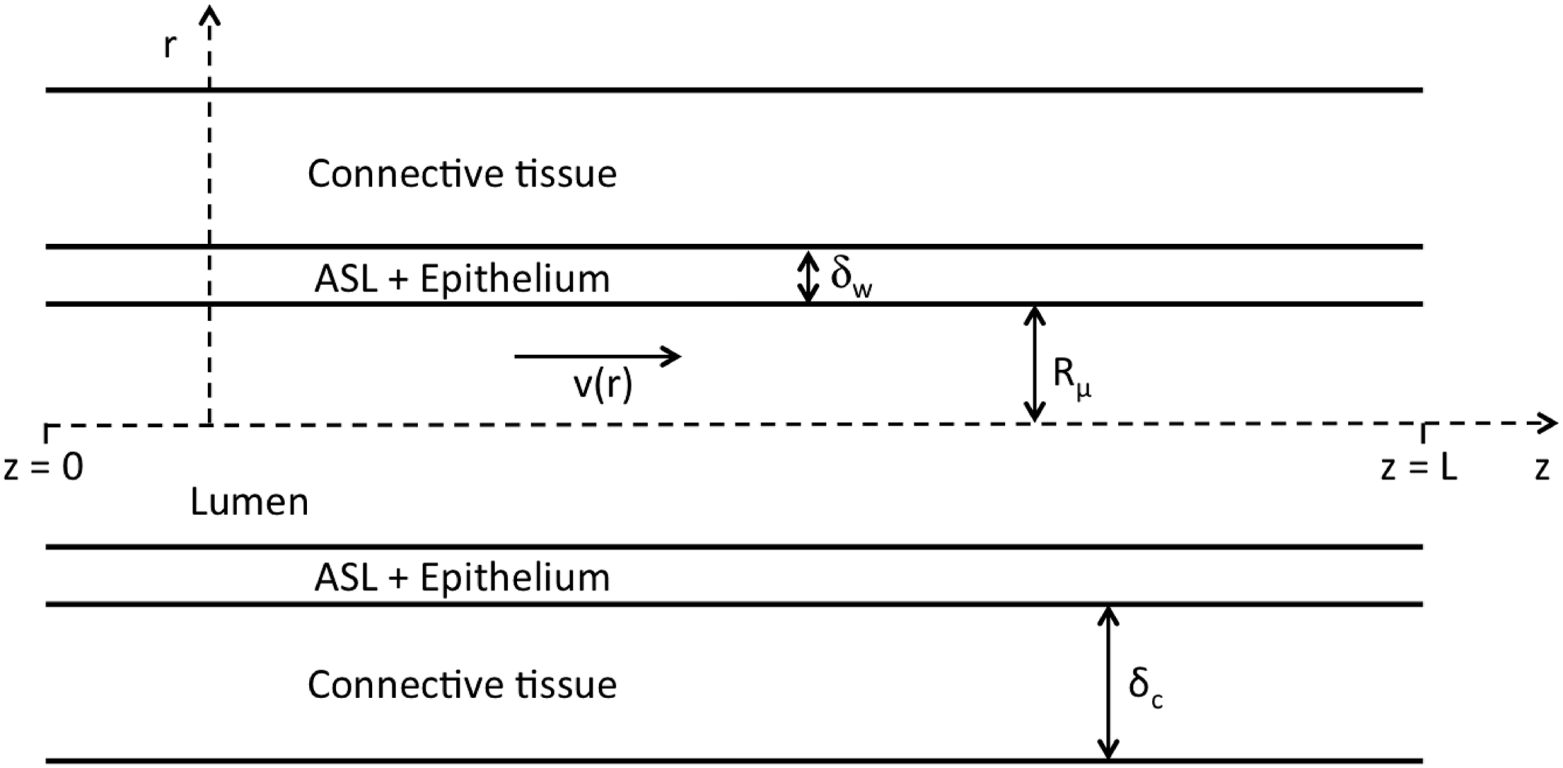
Schematic diagram of an airway, during inspiration. Several notations used in the model are presented on this figure. The picture is not to scale.

In this section, we present the computational tool designed to characterize the evaporation of the water contained in the mucus, in the bronchial region of the lungs. This computational tool is based on the expression of mass and energy transport equations in the lumen of the airways, in the ASL and in the epithelium. Originally, the model includes also a energy balance equation for the connective tissue surrounding the airway walls (see Fig 5).

The physicochemical properties appearing in the model are calculated using equations given in [57] and considering that the thermal properties of the ASL, the epithelium and the connective tissue surrounding the airways are close to those of liquid water. They are calculated at the body temperature *T*_*b*_ and, for the physicochemical properties of air, at a relative humidity of 1. This choice has very little influence on the calculations made. For instance, we have checked that using the temperature and the relative humidity of the air at the top of the trachea to evaluate the physicochemical properties does not lead to a significant change of the modeling results. This is due to the fact that, even in the case of breathing, at rest, a cold and dry air, the variations of the temperature and the relative humidity of the air in the bronchial region of the lungs remain limited (maximum 10°C for the temperature and maximum 0.2 for the relative humidity [27]). *C*_sat_(*T*) (mol m^−3^), the saturation concentration of water in air at the temperature *T*, is calculated using the Clausius-Clapeyron equation, with a reference temperature equal to *T*_*b*_, the temperature of the body (taken equal to 37°C).

As mentioned previously, we use a geometrical representation of the human lungs extensively described in a previous paper [31]. It is based on Weibel’s morphometric model [1]. We consider that all the airways are cylinders with axial symmetry and that all the airways belonging to the same generation have the same dimensions. Each airway belonging to generation *i* divides in two airways belonging to generation *i* + 1. This is called a bifurcation.

In the model, *r* and *z* axes are defined in each airway (see Fig 5). *r* is the radial coordinate (m), with *r* = 0 at the center of the airway and *r* = *R*_*μ*_ at the mucus–lumen interface. *R*_*μ*_ is assumed constant in a generation. *z* is the axial coordinate along the airway (m). This *z* axis is oriented in the direction of the flow during inspiration and in the opposite direction to the flow during expiration. *z* = 0 at one extremity of the airway and *z* = *L* at the other one. The average water concentration in the air entering the trachea during inspiration is RH^in^ *C*_sat_(*T*^in^). Generations 1 to 16 are considered in the model (generation 1 being the trachea). The air is assumed saturated with water and at body temperature beyond generation 16. Therefore, during expiration, the temperature of the air entering generation 16 is *T*_*b*_ and its average water concentration is *C*_sat_(*T*_*b*_). The validity of this assumption has been checked a posteriori and is demonstrated in other modeling works (see for instance [46]).

The equations of the model are presented for the inspiration phase only. Expiration is modeled in a similar way, except that the direction of the flow is reversed.

In the lumen of a single airway, unsteady transport of heat and water are considered. The temperature and concentration fields are assumed to be axisymmetric. Radial and axial diffusive transports of heat and mass are considered. The velocity field presented in Tawhai et al. [46] is used: $v\left(r\right)=v_{\text{av}}\frac{\gamma +2}{\gamma }\left(1-{\left(r/R_{\mu }\right)}^{\gamma }\right)$, where *v*(*r*) is the axial velocity (m s^−1^) at the radial coordinate *r*, with *v*_av_ the average velocity of the flow in the airway (m s^−1^). The radial velocity is assumed equal to zero. *γ* is a function of the Reynolds number of the flow in the airway (Re = 2*v*_av_ *R*_*μ*_/*ν*), where *ν* is the kinematic viscosity of the air (m s^−2^): *γ* = 2 for Re < 300 and *γ* = Re/150 for Re > 300. When *γ* = 2, this velocity field reduces to the one of a classical Poiseuille flow. For *Q*^in^ = 250 ml s^−1^ and for the geometrical model of the lungs considered in this study, it can be evaluated that such a flow is encountered from generation 6.

According to these assumptions, the balance equations for heat and mass transport in the lumen of the airway are:

$$\begin{array}{c} v_{\text{av}}\frac{\gamma +2}{\gamma }\left(1-{\left(\frac{r}{R_{\mu }}\right)}^{\gamma }\right)\frac{\partial C}{\partial z}+\frac{\partial C}{\partial t}=D\frac{1}{r}\frac{\partial }{\partial r}\left(r\frac{\partial C}{\partial r}\right)+D\frac{\partial^{2}C}{\partial z^{2}} \end{array}$$ (7)

$$\begin{array}{c} v_{\text{av}}\frac{\gamma +2}{\gamma }\left(1-{\left(\frac{r}{R_{\mu }}\right)}^{\gamma }\right)\frac{\partial T}{\partial z}+\frac{\partial T}{\partial t}=\alpha \frac{1}{r}\frac{\partial }{\partial r}\left(r\frac{\partial C}{\partial r}\right)+\alpha_{a}\frac{\partial^{2}C}{\partial z^{2}} \end{array}$$ (8)

where *t* is the time (s), *C*(*r*, *z*, *t*) is the local water concentration in the air (molm^−3^), *T*(*r*, *z*, *t*) is the local temperature of the air (K), *D* is the diffusion coefficient of water in air (m^2^s^−1^) and *α* is the thermal diffusivity of the humid air (m^2^s^−1^).

Actually, the velocity field in an airway might deviate from a velocity field with a zero radial velocity, for instance because recirculations are taking place at the bifurcations [58]. In order to take these recirculations into account, particular boundary conditions are used at the inlet of an airway (see below).

The transport of heat through the epithelium and the ASL is considered at quasi-steady state and purely radial. Indeed, in the epithelium and the ASL, the characteristic time of the radial diffusion of heat (a few hundredths of a second, considering a thickness of the epithelium of 50 *μ*m and a thickness of the ASL of 20 *μ*m) is way smaller than the inspiration time and than the characteristic time of the longitudinal diffusion of heat (dozens of second, even in small airways). Therefore, the following equation can be written to describe heat transport through the ASL and the epithelium:

$$\begin{array}{c} \frac{\alpha_{l}}{r}\frac{\partial }{\partial r}\left(r\frac{\partial T_{l}}{\partial r}\right)=0 \end{array}$$ (9)

where *T*_*l*_(*r*, *z*, *t*) is the temperature in the ASL and the epithelium and *α*_*l*_ is the thermal diffusivity of liquid water (m^2^s^−1^). This equation is written ∀*z* ∈ [0, *L*] and ∀*r* ∈ [*R*_*μ*_, *R*_*μ*_ + *δ*_*w*_], with *δ*_*w*_ the sum of the thickness of the ASL and the thickness of the epithelium (see Fig 5). *δ*_*w*_ is assumed constant. The results presented in this paper have been generated using a thickness of 20 *μ*m for the ASL and a thickness of 50 *μ*m for the epithelium (i.e. *δ*_*w*_ = 70 *μ*m). Actually, *δ*_*w*_ is expected to slightly decrease when the generation number increases. However, we have checked that the simulation results are very insensitive to the parameter *δ*_*w*_, mainly because the limitation to heat transfer is strongly located in the gas phase and not in the ASL and the epithelium.

In the connective tissue, the following energy balance is written (∀*z* ∈ [0, *L*]):

$$\begin{array}{c} \frac{dT_{c}}{dt}=\frac{P}{\rho_{l}c_{p,l}}-\frac{\alpha_{l}}{\delta_{c}}{\frac{\partial T_{l}}{\partial r}|}_{r=R_{\mu }+\delta_{w}} \end{array}$$ (10)

where *T*_*c*_(*z*, *t*) is the temperature of the connective tissue (K), *P* is the amount of heat produced in the connective tissue, per unit time and per unit volume (W m^−3^), *ρ*_*l*_ (kg m^−3^) and *c*_*p*,*l*_ (J kg^−1^ K^−1^) are the density and the heat capacity of liquid water, respectively, and *δ*_*c*_ is the thickness of the connective tissue involved in the heat transfer towards the lumen (m) (see Fig 5). *δ*_*c*_ is expressed as: $\delta_{c}=\sqrt{\alpha_{l}t^{\text{in}}}$. This equation is an original feature of the model. The second term of its right-hand side member characterizes the heat transferred from the connective tissue to the mucus–lumen interface. The first term of its right-hand side member characterizes the heat production in the connective tissue. This heat production is due to the renewal of the blood in the connective tissue. *P* is expressed as *P* = *β*(*T*_*b*_ − *T*_*c*_), with *β* a coefficient related to the blood flow in the connective tissue capillaries. To write this balance equation for the connective tissue, we have used the fact that *δ*_*w*_ and *δ*_*c*_ are small compared to *R*_*μ*_. An order of magnitude of *β* can be determined. Indeed, as *P* = *β*(*T*_*b*_ − *T*_*c*_) is the production of heat in the connective tissue surrounding the lungs, due to the blood flow, per unit time and per unit volume of this tissue, we can write that *β* ≃ *Q*_*b*_ *ρ*_*b*_ *c*_*p*,*b*_/*V*_*c*_, with *Q*_*b*_ the blood flow rate in the bronchial circulation (m^3^ s^−1^), *ρ*_*b*_ (kg m^−3^) and *c*_*p*,*b*_ (J kg^−1^ K^−1^) the density and the heat capacity of the blood, respectively, and *V*_*c*_ the volume of the connective tissue surrounding the bronchial region of the lungs (m^3^). Considering that the blood flows with a velocity *v*_cap_ ≈ 1 mm s^−1^ inside capillaries of diameter *d*_cap_≈ 8 *μ*m included in the connective tissue with a density *φ*_*c*_ of about 500 capillaries per square millimeter of connective tissue, it can be estimated that $Q_{b}/V_{c}=Q_{b}/S_{c}\times S_{c}/V_{c}\approx \phi_{c}\pi d_{\text{cap}}^{2}v_{\text{cap}}/4\times 1/\delta_{c}$, where $S_{c}$ is the area of the interface between the connective tissue and the epithelium. If we assume that the heat capacity and the density of the blood are close to those of liquid water and use *t*_in_ = 2 s, *β* ≃ 2 × 10^5^ W m^−3^ K^−1^ is calculated. This value of *β* has been used to generate all the results presented in this paper.

At the end of the airway (*z* = *L*, ∀*r* ∈ [0, *R*_*μ*_[), the axial diffusive fluxes are assumed to be equal to zero:

$$\begin{array}{c} \frac{\partial C}{\partial z}=0\;\text{and}\;\;\frac{\partial T}{\partial z}=0 \end{array}$$ (11)

At the center of the airway (*r* = 0, ∀*z* ∈ ]0, *L*[), axisymmetry is assumed:

$$\begin{array}{c} \frac{\partial C}{\partial r}=0\;\text{and}\;\;\frac{\partial T}{\partial r}=0 \end{array}$$ (12)

At the mucus–lumen interface (*r* = *R*_*μ*_, ∀*z* ∈ ]0, *L*[), the three following equations are written:

$$\begin{array}{c} T=T_{l} \end{array}$$ (13)

$$\begin{array}{c} -\lambda \frac{\partial T}{\partial r}+\lambda_{l}\frac{\partial T_{l}}{\partial r}=LD\frac{\partial C}{\partial r} \end{array}$$ (14)

$$\begin{array}{c} C=C_{\text{sat}}\left(T\right) \end{array}$$ (15)

where *λ* is the thermal conductivity of air (W m^−1^ K^−1^), *λ*_*l*_ is the thermal conductivity of liquid water (W m^−1^ K^−1^), L is the latent heat of vaporization of water (J mol^−1^). The first equation results from the continuity of the temperature at the interface, the second equation results from a heat balance at the interface and the third equation expresses that the air in contact with the mucus layer is saturated with water (as the molar fraction of mucins in the mucus is way smaller than one [10]).

At the interface between the epithelium and the connective tissue (*r* = *R*_*μ*_ + *δ*_*w*_, ∀*z* ∈ [0, *L*]), the continuity of the temperature is assumed:

$$\begin{array}{c} T_{l}=T_{c} \end{array}$$ (16)

The average water concentration and temperature at the outlet of the airway are evaluated as follows:

$$\begin{array}{c} C^{\text{out}}\left(t\right)=\frac{\int_{0}^{R_{\mu }}\left(1-{\left(\frac{r}{R}\right)}^{\gamma }\right)C\left(r,L,t\right)2\pi rdr}{\int_{0}^{R_{\mu }}(1-{\left(\frac{r}{R}\right)}^{\gamma })2\pi rdr} \end{array}$$ (17)

$$\begin{array}{c} T^{\text{out}}\left(t\right)=\frac{\int_{0}^{R_{\mu }}\left(1-{\left(\frac{r}{R}\right)}^{\gamma }\right)T\left(r,L,t\right)2\pi rdr}{\int_{0}^{R_{\mu }}(1-{\left(\frac{r}{R}\right)}^{\gamma })2\pi rdr} \end{array}$$ (18)

In an airway belonging to generation *i*, $v_{\text{av}}=Q^{\text{in}}/\left(2^{i-1}\pi R_{\mu_{i}}^{2}\right)$, with *R*_*μ*_*i*__ the value of *R*_*μ*_ for the generation i. In the trachea, *T*(*r*, *z* = 0, *t*) = *T*^in^ and *C*(*r*, *z* = 0, *t*) = RH^in^ *C*_sat_(*T*^in^). At the inlet of an airway belonging to generation *i*, $T\left(r,z=0,t\right)=T_{i-1}^{\text{out}}\left(t\right)$ and $C\left(r,z=0,t\right)=C_{i-1}^{\text{out}}\left(t\right)$, where $T_{i-1}^{\text{out}}\left(t\right)$ and $C_{i-1}^{\text{out}}\left(t\right)$ are the averaged temperature and water concentration at the outlet of an airway belonging to generation *i* − 1 (evaluated using Eqs (17) and (18)). This homogenization of the concentration and temperature profiles at the inlet of an airway is imposed in order to take into account the recirculations of the flow induced by the bifurcations.

In order to simulate an inspiration phase, the equations written above are solved successively for each generation, starting with the trachea. To simulate an expiration phase, the equations describing heat and mass transports during expiration are also solved successively, but starting with generation 16. From a given initial situation, several cycles can be simulated consecutively, until the temperature profile of the connective tissue obtained at the end of a cycle is equal (with a certain tolerability) to that obtained at the end of the previous cycle. Then, for a given airway, *E*, the ratio between the volume of liquid water evaporated in the airway during the cycle and the duration of the cycle (m^3^ of liquid water per second) is evaluated as follows:

$$\begin{array}{c} E=\frac{M_{w}}{\rho_{l}}\frac{1}{t^{\text{in}}+t^{\text{ex}}}\int_{0}^{t^{\text{in}}+t^{\text{ex}}}\int_{0}^{L}2\pi R_{\mu }D{\frac{\partial C}{\partial r}|}_{r=R_{\mu }}dzdt \end{array}$$ (19)

with *M*_*w*_ (kg mol^−1^) the molar mass of water.

The equations of this model are solved in two steps.

First, Eq (9) is solved analytically. Using Eqs (13) and (16), the unknown constants appearing in the general solution of this equation can be expressed as functions of *T*_*c*_(*z*, *t*) and the local temperature of the air at the mucus–lumen interface *T*(*R*_*μ*_, *z*, *t*). ∂*T*_*l*_/∂*r* at the mucus–lumen interface and at the connective tissue–epithelium interface are then locally expressed as functions of these two temperatures and introduced in Eqs (10) and (14).

Second, for given conditions (*Q*^in^, *t*^in^, *t*^ex^, *T*^in^ and HR^in^), the equations of the model are solved numerically, in a dimensionless form and after spatial discretization, using the finite difference method, with the NDSolve function of Wolfram Mathematica 10. 50 discretization intervals are used in the longitudinal direction, while 70 intervals are used in the radial direction. It has been checked that these numbers are large enough for the simulation results to be independent of the size of the grid. The convection terms in Eqs (7) and (8) are discretized using a first order upwind scheme and the diffusion terms are discretized using a second order centered scheme. The derivatives in Eqs (11), (12) and (14) are discretized with a first order scheme. The integrals in Eqs (17) and (18) are evaluated using the trapezoidal rule. The derivative in Eq (19) is discretized using a second order scheme, the integral over *z* is evaluated using the trapezoidal rule and the integral over time is evaluated using the NIntegrate function of Wolfram Mathematica 10. Finally, Eq (15) is linearized around *T* = *T*_*b*_. It has been checked that this linearization has no significant influence on the simulation results.
